# Inflammation, Aging and Hematopoiesis: A Complex Relationship

**DOI:** 10.3390/cells10061386

**Published:** 2021-06-04

**Authors:** Pavlos Bousounis, Veronica Bergo, Eirini Trompouki

**Affiliations:** 1 Department of Cellular and Molecular Immunology, Max Planck Institute of Immunobiology and Epigenetics, 79108 Freiburg, Germany; bousounis@ie-freiburg.mpg.de (P.B.); bergo@ie-freiburg.mpg.de (V.B.); 2 Faculty of Biology, University of Freiburg, 79104 Freiburg, Germany; 3 International Max Planck Research School for Immunobiology, Epigenetics and Metabolism (IMPRS-IEM), 79108 Freiburg, Germany; 4 Centre for Integrative Biological Signaling Studies (CIBSS), University of Freiburg, 79104 Freiburg, Germany

**Keywords:** hematopoiesis, hematopoietic stem cells, bone marrow niche, regeneration, aging, inflammatory signaling, myeloid bias, myelodysplasia

## Abstract

All vertebrate blood cells descend from multipotent hematopoietic stem cells (HSCs), whose activity and differentiation depend on a complex and incompletely understood relationship with inflammatory signals. Although homeostatic levels of inflammatory signaling play an intricate role in HSC maintenance, activation, proliferation, and differentiation, acute or chronic exposure to inflammation can have deleterious effects on HSC function and self-renewal capacity, and bias their differentiation program. Increased levels of inflammatory signaling are observed during aging, affecting HSCs either directly or indirectly via the bone marrow niche and contributing to their loss of self-renewal capacity, diminished overall functionality, and myeloid differentiation skewing. These changes can have significant pathological consequences. Here, we provide an overview of the current literature on the complex interplay between HSCs and inflammatory signaling, and how this relationship contributes to age-related phenotypes. Understanding the mechanisms and outcomes of this interaction during different life stages will have significant implications in the modulation and restoration of the hematopoietic system in human disease, recovery from cancer and chemotherapeutic treatments, stem cell transplantation, and aging.

## 1. Introduction

The establishment and maintenance of the lifelong supply of blood cells relies on a rare population of multipotent and self-renewing hematopoietic stem cells (HSCs), which reside in specialized niches within the bone marrow (BM) [1]. The hematopoietic process, from development to adulthood, as well as the genetic programs regulating HSC biology are well conserved among vertebrates [1,2,3]. Embryonic hematopoietic development proceeds via distinct waves. The first or “primitive” wave is HSC-independent and gives rise to hematopoietic cells required for the immediate needs of the developing embryo, such as erythrocytes, megakaryocytes, myeloid cells, and macrophages. The intermediate wave gives rise to transient erythro-myeloid precursors. During the third “definitive” wave, HSCs emerge from the aorta-gonad-mesonephros (AGM) region of the developing embryo, specifically from hemogenic endothelial cells located in the dorsal aorta, via an endothelial-to-hematopoietic transition (EHT). The newly generated HSCs then migrate to the fetal liver, where they expand before reaching the BM, the site of adult hematopoiesis. Once HSCs localize to the BM, the vast majority remain quiescent within specialized niches [1,4,5,6].

The maintenance of HSCs in a non-cycling, quiescent state keeps energy requirements low, prevents oxidative damage, and ensures lifelong availability of a stable pool of viable HSCs that can be activated as needed to maintain and restore the hematopoietic system [7]. HSC quiescence, self-renewal, and differentiation are controlled by cell-intrinsic mechanisms, mainly involving transcriptional, epigenetic and metabolic alterations, but also cell cycle regulators [8], as well as cell-extrinsic signals from the cells and extracellular matrix (EM) of the BM niche [9,10,11]. The self-renewal capacity of HSCs progressively diminishes as long-term-HSCs (LT-HSCs) differentiate into short-term-HSCs (ST-HSCs), and eventually multipotent progenitors (MPPs), but is also affected by “stress” signals such as infections, pathological conditions or physiological stress such as aging [12,13,14]. It is critical to understand how HSC homeostasis is preserved under steady-state conditions and how it is affected by stress conditions, as its deregulation may result in the development of hematological malignancies.

Inflammation has evolved as an adaptive response against infection, tissue injury, and other “stress” signals, used to induce regeneration and restore tissue homeostasis via the removal of damaged cells and stem cell activation. The relationship between inflammation and HSCs has been the focus of many recent studies [9,15,16]. Inflammatory signaling is a critical regulator of HSC development [17,18,19,20,21,22,23] and maintenance during adulthood. In fact, adult, quiescent HSCs are rapidly activated in response to inflammatory signals, which affect not only their proliferation rate, but also their commitment towards specific lineages [24,25,26,27,28,29]. Systemic, low-grade chronic inflammation has been shown to affect HSC functionality, numbers, and differentiation, but is also one of the hallmarks of the aged hematopoietic system [30,31,32]. Excessive exposure to these signals can also be a harbinger of HSC exhaustion and functional impairment [33]. Moreover, it has been accepted that HSC aging entails increased numbers of functionally impaired HSCs with reduced self-renewal, and a bias towards myeloid differentiation [7,30,31,34,35].

In this review, we outline the known effects of inflammation on HSCs and their niche during adulthood and upon aging. Understanding how inflammation affects HSC homeostasis and cell fate decisions can lead to the development of clinical interventions and therapies for maintaining a healthy hematopoietic system after insults, cancer treatments, and aging.

## 2. Inflammatory Signaling as a Key Regulator of HSC Homeostasis

HSC quiescence and activation are thoroughly modulated by a complex interplay between cell-intrinsic and cell-extrinsic factors. Over the past years, many studies have investigated the role of inflammatory signaling in HSC homeostasis, proposing inflammation as a key regulator of HSC fate.

Interferons (IFNs) are a potent family of autocrine and paracrine cytokines that include Type I IFNs: IFN-α, IFN-β and others, or Type II IFNs: IFN-γ. They are fundamental for the mediation and resolution of infections [36]. HSCs are able to sense and respond to IFNs, as they express a plethora of different IFN receptors on their cellular surface. While low steady-state levels of IFNs may be required for HSC homeostasis, acute or chronic IFN exposure can have deleterious effects on the HSC compartment, as hyperstimulation by IFNs leads to HSC overactivation and eventually exhaustion [37]. Exposure to IFN-α forces HSCs to exit quiescence in a STAT1-dependent manner, ultimately leading to self-renewal defects [25]. HSCs need to return to a quiescent state in order to prevent exhaustion, and they appear to employ not fully characterized “braking” mechanisms. For example, exposure to Type I IFNs transiently induces HSC activation *in vivo*, followed by re-entry into quiescence if IFN exposure persists chronically [26], a mechanism that has been suggested to be c-MYC-dependent [38]. In this context, the cell cycle machinery is not affected by IFNs. Instead, IFNs trigger a brief, but broad transcriptional downregulation of many quiescence-enforcing genes, such as *Foxo3a*, *Foxo1*, and *Pten*, as well as Notch and TGF-β associated genes, to a level that allows HSC proliferation. Other cell-intrinsic mechanisms can modulate the activation of IFN response as well. For example, IRGM1 and the RNA-editing enzyme ADAR1 have been identified as critical feedback inhibitors, which balance and prevent the deleterious effects of prolonged IFN exposure. IRGM1 deficient HSCs exhibit elevated IFN signaling accompanied by hyperproliferation, self-renewal and autophagy defects which are normalized in the absence of IFN receptors or STAT1 [39]. ADAR1 is an essential regulator of the IFN response in HSCs, as its deficiency leads to a widespread upregulation of transcripts associated with Type I and Type II interferon responses, triggering HSC pool exhaustion [40]. The effects of IFNs on HSCs, however, appear to be context-specific. IFN-γ, for instance, can either stimulate or suppress HSC proliferation and reconstitution capacity. IFN-γ produced during bacterial infections transiently activates HSCs to exit dormancy and differentiate, yet prolonged IFN-γ exposure in chronic infections appears to negatively affect the maintenance of mouse HSCs, decreasing the number of self-renewing divisions favoring asymmetric divisions, reducing engraftment, and leading to HSC exhaustion. HSC cell-cycle entry, proliferation, differentiation, and apoptosis, however, were not impeded, which is consistent with the activation-to-exhaustion effects of other pro-inflammatory cytokines [24,41,42,43]. Interferons produced in response to infection also induce megakaryopoiesis. It was shown by Haas et al. that a lineage-primed population of HSCs designated stem-like megakaryocyte-committed progenitors (SL-MkPs) rapidly restores platelets after infection [44].

Tumor necrosis factor α (TNFα) acts as a pro-inflammatory cytokine in a variety of processes, from fever stimulation to the regulation of tissue homeostasis, by influencing cell proliferation and differentiation [45,46,47,48]. Like with interferons, the contribution of TNFα signaling to HSC homeostasis depends on the dose and duration of the exposure, and could be influenced by the local environment within the BM [49]. TNFα exposure in vitro has been reported to negatively regulate the maintenance of cycling HSCs by promoting differentiation at the cost of self-renewing divisions [50,51]. In addition, HSCs exposed to TNFα were not able to sustain multi-lineage differentiation upon transplantation into NOD-SCID mice, and exhibited skewing towards myeloid differentiation [50]. However, it has also been demonstrated that in vitro TNFα production by CD8+ T-cells strengthens HSC function and supports hematopoietic reconstitution upon transplantation, as HSCs showed a better engraftment capacity [52]. In vivo experiments have also proven that TNFα overexpression restricts the activity of HSCs by a mechanism that depends on TNFRS1a and TNFRS1b receptors. This effect seems to affect mainly cycling rather than quiescent HSCs, and it can change with age [53]. At the molecular level, TNFα activates a NF-κB-dependent transcriptional program that promotes HSC survival and poises them for myeloid differentiation [54] through the upregulation of PU.1, a myeloid lineage-instructing transcription factor [55].

Studying the effects of interleukins (ILs) on HSC homeostasis is complicated, as they have often redundant functions. Interleukins play a plethora of essential roles within the immune system, such as sustaining proliferation, maturation and migration. Moreover, they have both pro- and anti-inflammatory properties, according to the microenvironment and the cell types they act on [56]. The effects of interleukins on adult HSCs are still unclear. Acute IL-1 exposure is known to act as a “pro-inflammatory” signal during infection, promoting cell division and priming HSCs towards myeloid differentiation through the activation of the transcription factor PU.1 [27]. Chronic exposure strongly impairs HSC self-renewal capacity, as they fail to replenish the hematopoietic system upon transplantation [27]. However, these results have been proven to be transient. Recent results from the same group showed that the exposure of LT-HSCs to IL-1 activates PU.1 that directly binds and represses genes related to cycling and protein synthesis, thus safeguarding HSC functionality [57]. In addition, IL-27 has been shown to induce the expansion of the hematopoietic stem and progenitor cell (LSK) compartment during emergency hematopoiesis, promoting myeloid differentiation [58]. Among the others, IL-33 has been studied in the context of total body irradiation, showing that it enhances HSC survival, preventing the activation of apoptosis by repressing the TP53-PUMA pathway [59]. Additionally, IL-12 has been shown to support HSC self-renewal in vitro by controlling the division rate through the activation of the JAK/STAT pathway [60]. Finally, IL-6 produced after the stimulation of Toll-like receptors (TLRs) is important for myeloid differentiation and hematopoietic stem and progenitor cell (HSPC) proliferation [61].

The function of TLRs is also important for HSCs. Agonists against TLR7/8 induce the expansion and mobilization of HSPCs [62], while germline or mosaic activation of TLR8 leads to immunodeficiency and bone marrow failure [63]. The function of the granulocyte colony-stimulating factor (G-CSF) in HSCs is also mediated, at least partly, by TLR2/4 [64], while pathogen-induced TLR4 signaling leads to HSC cycling, but at a loss of self-renewal capacity [65].

In conclusion, inflammation plays a pivotal role in “jump-starting” dormant HSCs in response to infection or hematological insults or when “emergency hematopoiesis” is needed. Inflammatory signals skew HSC differentiation towards first-responder cells of the myeloid lineage. If the inflammatory response is not resolved and chronically persists, the overall effect on HSCs can lead to their exhaustion.

## 3. Inflammation Affects HSC Homeostasis via the BM Niche

The BM niche has emerged in the last few years as an important regulator of HSCs even though the exact localization of HSCs in the BM niche is still under debate. The structure of the BM niche is complex and involves multiple cell types that regulate not only HSCs but also distinct progenitor populations. The BM mesenchymal stromal cells (MSCs) are rare perivascular cells important for HSC maintenance together with other cells such as osteoblasts, endothelial cells, and neuronal cells, but also some hematopoietic cells such as megakaryocytes and macrophages [66,67,68].

Inflammation influences HSCs in a cell-extrinsic manner by altering their BM niche, as shown by a growing number of studies [67,69,70]. BM niche cells respond to peripheral inflammation or infection by releasing factors that promote myelopoiesis [71,72]. For example, peripheral inflammation drives the secretion of G-CSF, mainly from endothelial cells, which then plays a central role in the differentiation of hematopoietic progenitors towards mature granulocytes [71,73]. Inflammation also affects the proximity of HSCs to quiescence-promoting BM niche cells, which is also an important factor affecting HSC functions. Under homeostatic conditions, CXCR4 expressed by HSCs interacts with CXCL12 on CXCL12-abundant reticular (CAR) cells residing in the HSC niche [74,75]. The stimulation of HSCs with IFN-γ in culture causes their displacement from quiescence-promoting CAR cells. This displacement was shown to occur via upregulation of the expression of the glycoprotein BST2 on the HSC surface [28]. Additionally, it was shown that IFN-γ secreted by cytotoxic CD8+ T cells in a model of lymphocytic choriomeningitis virus infection stimulates MSCs to secrete IL-6, which in turn enhances myelopoiesis [76]. Integrin signaling synergizes with IFN-γ to modulate HSCs. Indeed, integrin β3 signaling enhances STAT-1 mediated signaling upon IFN-γ treatment and thus intensifies the effect of IFN-γ on HSCs [77]. Type-I IFNs influence HSCs by affecting the BM niche as well. Prednergast et al. demonstrated that the BM of IFN-α or poly(I:C)-treated mice exhibited increased vascularity, expansion, and activation of endothelial cells. Mechanistically, these changes were mediated by vascular endothelial growth factor (VEGF) signaling crosstalk between ECs and other BM cells, including HSCs. This facilitated BM remodeling and suggests a role for HSCs themselves in the regulation of their niche [78]. Moreover, BM granulocytes secrete TNFα, which acts on ECs and promotes vessel and hematopoietic regeneration [79]. This effect was lost in mice deficient for TNF receptors, and transplantation experiments proved the direct function of TNFα on HSCs. Recently, single cell expression analysis showed that poly(I:C) or lipopolysaccharide (LPS) administration induces abundant cytokine expression from CAR cells (IL6, IL1b, CXCL2 and CXCL5) and sinusoidal endothelial cells concomitant to the downregulation of factors that contribute to HSC retention and lymphopoiesis, thus providing a mechanistic explanation for the observed myeloid skewing [72].

These studies show that inflammation can affect HSC activity via niche-dependent interactions. However, how inflammation directly affects specific populations in the BM niche and how they consequently affect HSCs need further investigation.

## 4. Inflammation and HSC Aging

**Figure 1:**
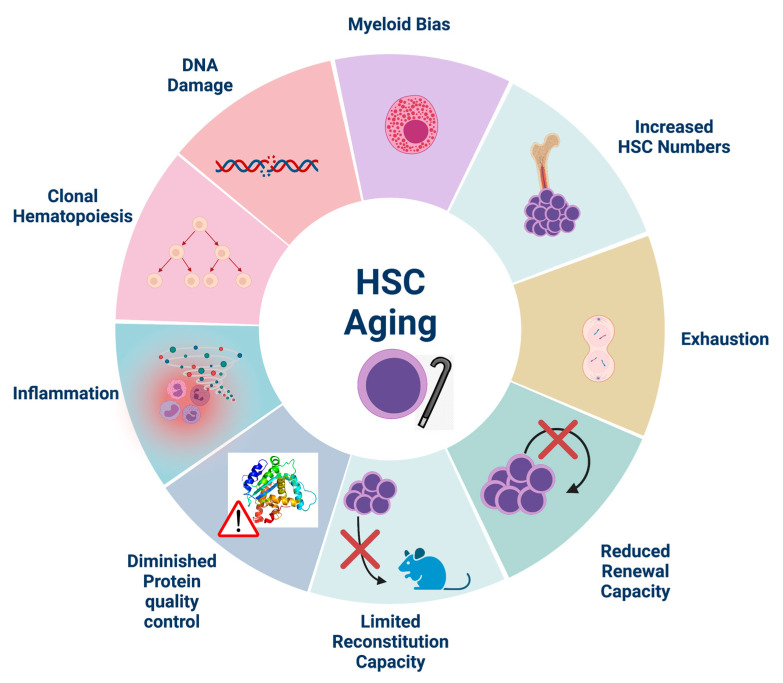
Hallmarks of HSC aging. Aged HSCs exhibit diminished self-renewal and reconstitution capacity upon transplantation which contributes to HSC exhaustion despite their increased numbers in aged individuals. They are also characterized by myeloid skewing, DNA damage accumulation and decreased protein quality control. Inflammation, although essential for HSC homeostasis, is increased during aging and exerts detrimental effects on aged HSCs. Indeed, inflammation accelerates the proliferation of mutated HSCs, thus contributing to clonal hematopoiesis. Created with BioRender.com, accessed on 29 May 2021.

Although the number of HSCs residing in the BM is generally increased in aged individuals, aged HSCs exhibit impaired long-term repopulation capacity [80] (Figure 1). One of the most prominent characteristics of aged HSCs is “inflamm-aging”, defined as a sterile, systemic, low-grade chronic inflammatory phenotype [29]. An increasing number of publications indicate that chronic inflammation influences HSC functionality, differentiation, and renewal in aged organisms both directly and indirectly [29,81]. The exact mechanisms of how these changes occur in HSCs and the role of “inflamm-aging” in this process are still being elucidated.

Increased senescence accompanied by the secretion of pro-inflammatory cytokines such as IL-1, TNFα, and IL-6 and others, immune modulators, growth factors, and proteases—termed the senescence-associated secretory phenotype (SASP)—accompanies and most likely plays a role in diminishing the functionality of aged HSCs [29,81,82,83]. A recent study showed that age-related inflammation promotes HSC aging by inducing the surface expression of IL27Ra via TNFα-ERK-ETS1 signaling. The deletion of IL27Ra was shown to rescue the functional decline and myeloid bias of HSCs by reversing the effects of TNFα [81]. Likewise, endogenous activation of NF-κB signaling mediated by RAD21/cohesin in aged mice led to increased responsiveness to inflammation, consequently driving HSC differentiation and leading to the loss of their self-renewal capacity [84]. As with TNFα, increased exposure to IL-1 also biases aged HSCs towards myeloid differentiation via NF-κB-mediated activation of PU.1, likely signifying a common downstream response of HSCs to pro-inflammatory signals that become more active with age [29]. Results from another study showed that the P38a isozyme of P38-MAPK differentially influences HSC differentiation bias and re-population capacity in young versus aged cells. The activation of P38-MAPK signaling in response to stress, DNA damage, inflammatory signaling, BM transplantation or loss of ATM induces reactive oxygen species (ROS), leading to decreased HSC quiescence and exhaustion [85]. Mann et al. identified a CD61 positive population of LT-HSCs that responds to inflammatory challenges and is potentially responsible for the myeloid bias observed in aged HSCs [86], in accordance with previous studies showing that functionally distinct HSCs are responsible for the myeloid bias [87].

HSCs naturally accumulate DNA damage as they age, which significantly contributes to age-related tissue degeneration and malignancies [88,89,90]. The extent of DNA damage is directly associated with the replicative stress induced in HSCs as they enter the cell cycle in response to external cues, and depends, at least in part, on the effects of IFN-α on LT-HSCs. DNA damage in HSCs was shown, however, to most likely be a result of cell cycle entry and not of an IFN-α-induced transcriptional program [89]. In addition, repeated activation and re-entry into quiescence of HSCs in adult WT mice via serial activation of type-I IFN response with poly(I:C) led to a progressive depletion of functional HSCs, with no sign of later recovery due to the absence of self-renewal divisions [91]. High levels of replicative stress during aging are associated with cell cycle defects and chromosomal abnormalities, mainly due to decreased expression of MCM helicase components [88].

Dysfunction of the HSC epigenetic regulatory machinery has been shown to play a prominent role in the chronically inflamed aged phenotype as well. A recent study by Sera et al. [92] demonstrated the role of the epigenetic modifier ubiquitously transcribed tetratricopeptide repeat gene, X chromosome (UTX/KDM6A) in maintaining HSC “youthfulness”. UTX levels in HSCs are known to decrease with age, and HSPCs from UTX-deficient mice displayed an aged gene expression program, including impaired DNA repair mechanisms, ROS accumulation, and increased expression of the aging-associated marker CD41. UTX-deficient mice further presented with trilineage dysplasia, a condition similar to myelodysplastic syndrome (MDS) in humans, which becomes more common with age [92]. It is possible that these aging effects on HSCs are caused by inflammation, as UTX has been associated with deregulated inflammatory signaling in a UTX-deficient mouse model of bladder cancer [93].

Of note, chaperone-mediated autophagy (CMA) has been shown to maintain adult HSC function in mice by controlling protein quality and up-regulating fatty acid metabolism in active HSCs. Quiescent HSCs from young mice had higher levels of CMA activity compared to quiescent HSCs from older mice, and CMA activity was further increased in the activated HSCs after 5-FU treatment [94]. Importantly, CMA has been shown to decrease in response to inflammation, and reduced CMA activity contributes to the aged HSC phenotype [81,94]. Reactivating CMA alleviated some of the phenotypes of aged HSCs [94]. In general, autophagy has been shown to be critical for maintaining the metabolism and function of both young and old HSCs [95].

While several studies pinpoint a role of inflammatory signaling in hematopoietic aging, it is still debatable whether we know the exact cytokine profile important for HSC aging and the interplay between different sterile inflammatory signals.

## 5. Inflammation Alters the Aged HSC Niche

**Figure 2:**
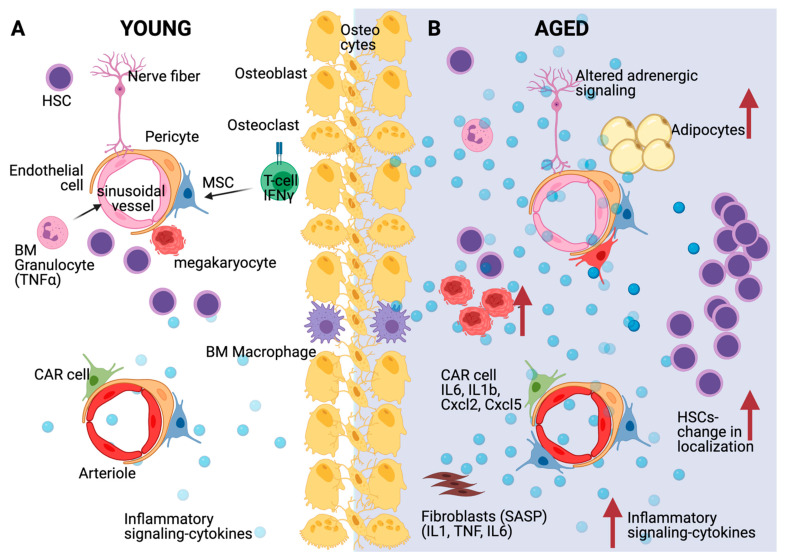
Effects of inflammation and aging on the HSC niche. (**A**) Multiple bone marrow niche cells such as mesenchymal stromal cells (MSCs) and specifically CXCL12-abundant reticular (CAR) cells, endothelial cells, but also hematopoietic cells such as megakaryocytes, T-cells and macrophages affect HSC quiescence through diverse molecular mechanisms, including cytokine secretion as depicted. (**B**) During aging, HSC accumulation but with increased distance from arterioles, sinusoids and megakaryocytes is accompanied by inflammatory signaling and cytokine secretion from various bone marrow populations including CAR cells and senescent fibroblasts (SASP: Senescence-associated secretory phenotype). Aged MSCs turn primarily into adipocytes and adrenergic signaling is disturbed. Created with BioRender.com, accessed on 3 June 2021.

HSCs are influenced by age-related changes to the BM niche, and especially by the elevated levels of pro-inflammatory cytokines expressed in the aged niche (Figure 2). The inflammatory milieu in the aged bone marrow drives stromal and hematopoietic remodeling. Specifically, osteogenic MSCs decrease in numbers at the endosteum while pro-inflammatory perisinusoidal MSCs expand in the central marrow. Importantly, blocking IL-1 signaling improves the features of the aged hematopoietic system [96]. Ergen et al. showed that the production of the cytokine CCL5 by BM niche cells is increased with age and is pivotal for the myeloid skewing observed in aged HSCs [97]. The overexpression of CCL5 in young mice mirrored the decrease in lymphoid and increase in myeloid lineage production observed in aged mice, while *Ccl5* knockout HSCs exhibit decreased myeloid bias [97]. Mature myeloid and megakaryocytic cells themselves are known sources of inflammatory cytokines, which likely reinforce and perpetuate the myeloid skewing of HSCs with age [98]. Indeed, increased inflammatory signals concomitant to decreased phagocytosis from macrophages instigate a platelet bias in the aged BM [99]. TNFα produced by macrophages, lymphocytes, and endothelial cells regulates HSC gene expression via activation of the transcription factor NF-κB, and exerts diverse effects, especially during aging [54].

Single-cell transcriptome analysis of the mouse BM niche after treatment with 5-fluorouracil, a myeloablative chemotherapeutic agent, showed significant transcriptional remodeling within the BM populations. These changes include the expansion of the perivascular population, and the downregulation of the Notch delta-like ligands *Dll1* and *Dll4*, which promote a myeloid-differentiation transcriptional program in HSCs [83]. Furthermore, aging induces the gradual replacement of BM cells, such as stromal and perivascular cells, with adipocytes, which are a source of regulatory cytokines. When MSCs differentiate into adipocytes, they cease to produce and secrete factors important for hematopoiesis and HSC maintenance, such as CXCL-12, IGF-1, and c-KIT ligand. These alterations mirror chronically inflamed, aged hematopoietic systems [37]. Adrenergic signaling is also important for aged HSCs [100]. Increased sympathetic adrenergic activity has been previously described during aging [101]. Recently, it has been shown that aging remodels the HSC niche by expanding non-endosteal neurovascular niches at the expense of endosteal niches. In addition, increased β2-adrenergic signaling during aging promotes IL-6 dependent myeloid differentiation [102].

These studies show that sterile inflammation is important for the remodeling of the aged bone marrow niche, which in turn affects HSCs.

## 6. Inflammation, Aging and Hematological Disorders

Inflammation is an acute response to various stress conditions and often leads to restoration of tissue homeostasis. However, failure in resolving inflammatory cues may impair tissue function. Given the role of inflammation in HSC homeostasis, it is not surprising that the deregulation of this interaction affects the emergence, progression, and outcome of various hematological disorders. Indeed, the connection between deregulated inflammatory signaling and bone marrow dysfunction has been extensively described.

Myelodysplastic syndromes (MDS) are a heterogeneous group of hematological disorders, characterized by defective hematopoiesis, blood dysplasia, and the tendency to progress to leukemia [103]. In the past years, further evidence supporting the role of inflammatory signaling dysregulation in MDS has been presented [104,105], demonstrating that the BM microenvironment also plays a critical role in disease emergence and progression [106]. For example, MDS patients show increased levels of IFN-γ and TFNα in the bone marrow [107], which is associated with higher apoptosis rates in MDS BM cells [108]. Multiple components of the TLR signaling pathway, such as receptors and downstream mediators, were found to be overexpressed or altered in MDS [109]. For instance, increased expression of TLR2, TLR4 and TLR9 has been correlated with higher levels of TFNα, which decreases upon leukemic transformation [110,111]. MDS progression leads to the development of leukemia, a broad group of either acute or chronic hematological malignancies. In particular, the progression of MDS to acute myeloid leukemia (AML) is observed in one third of the patients [112].

Over the past years, inflammation has been directly linked to myeloid malignancies and proposed as one of the drivers of pathogenesis, while also influencing disease progression and burden [113,114]. The NF-κB pathway plays an important role in HSC proliferation and differentiation, and as expected it was shown to be constitutively active in AML cells [115]. IFN-γ and TFNα have been shown to promote differentiation and suppress clonal growth in AML blasts [116]. A recent study has also described a crucial contribution of the microenvironment to AML. Indeed, inflammatory cytokines, such as IL-6 and IL-1β, released by mesenchymal and progenitor cells are sensed by AML cells, activating JAK/STAT signaling to boost AML progression [117]. Elevated cytokines levels are also observed in patients with myeloproliferative neoplasms (MPN) [118] and chronic myeloid leukemia (CML) [119].

Inflammation also contributes to clonal hematopoiesis (CH). As HSCs age, they are more likely to accumulate DNA damage and mutations as a consequence of both time and impaired DNA repair mechanisms, leading ultimately to hematological disorders. If an HSC gains at least one mutation that grants it a selective advantage, it may give rise to a population of mutated clones. CH occurs when this altered HSC population ends up significantly contributing to the total number of mature blood cells. Clonal hematopoiesis of indeterminate potential (CHIP) describes the progression of CH to a level where at least 4% of all nucleated blood cells harbor a cancer-associated mutation, and this condition can progress to leukemia [120]. For example, mutations in genes encoding for epigenetic regulators such as *Dnmt3a, Tet2,* and *Asxl1* confer an advantage in terms of self-renewal and inhibit differentiation [121]. In vitro exposure of TET2-deficient human and murine HSCs to TNFα leads to a strong proliferative advantage [122], while mouse TET2-deficient HSCs resist apoptosis under inflammatory stress and increase their repopulation capacity [123]. Recently, it has been shown that inflammation could drive the expansion of *Dnmt3a* knockout cells, thus mimicking clonal hematopoiesis [124]. Additional studies need to be performed, as new insights on clonal hematopoiesis can enhance our understanding on preleukemic development and cancer evolution. Moreover, understanding the features of CH will also help predict and prevent the progression of this condition to hematological malignancies.

Inflammation also plays a role in genetically inherited conditions. Fanconi anemia (FA) is characterized by genomic instability, bone marrow failure, short stature and a high relative risk of myeloid leukemia. FA stem cells are especially vulnerable to a variety of inflammatory stress signals [89,125]. FA has been associated with increased bone marrow levels of IFN-γ and TFNα [126], and another study showed that although acute TNFα exposure profoundly inhibited the growth of *Fancc^−/−^* stem cells, chronic exposure promoted leukemic clonal evolution within the HSC compartment [127].

The interplay between inflammation and hematological disorders is evident. Future studies will help to better dissect the contribution of inflammatory signaling in disease progression.

## 7. Discussion

Inflammation appears to act as a multi-edged sword in the context of hematopoiesis. Although certain levels of inflammation are required for proper HSC development, maintenance, activation, and differentiation, sustained chronic or excessive levels of inflammatory signaling negatively affect the function and self-renewal of HSCs, and bias their differentiation program. In addition to the cell-intrinsic effects of inflammatory signaling of HSCs, there is a growing body of evidence that inflammatory signals from the BM microenvironment also contribute to HSC aging. Understanding the mechanisms of inflammation during adulthood and upon aging will have important clinical implications in regenerative therapies, stem cell transplantation and the treatment of leukemias. Pinpointing which pathways to modulate in aged stem cells could improve cancer treatment outcomes at all ages and make the transplantation and engraftment of HSCs from older individuals feasible, thereby significantly expanding the pool of potential donors.

## Abbreviations

AGM	Aorta-Gonad-Mesonephros
AML	Acute Myeloid Leukemia
BM	Bone Marrow
CH	Clonal Hematopoiesis
CHIP	Clonal Hematopoiesis of Indeterminate Potential
CMA	Chaperone-Mediated Autophagy
CML	Chronic Myeloid Leukemia
FA	Fanconi Anemia
G-CSF	Granulocyte Colony-Stimulating Factor
HSC	Hematopoietic Stem Cell
HSPC	Hematopoietic Stem and Progenitor Cell
IFN	Interferon
IL	Interleukin
LPS	Lipopolysaccharide
LT-HSC	Long Term Hematopoietic Stem Cell
MDS	Myelodysplastic Syndromes
MPN	Myeloproliferative Neoplasms
MPP	Multi-Potent Progenitor
MSC	Mesenchymal Stem Cell
SASP	Senescence-Associated Secretory Phenotype
ST-HSC	Short Term Hematopoietic Stem Cell
TLR	Toll-like Receptor
TNFα	Tumor Necrosis Factor α
VEGF	Vascular endothelial growth factor
